# Putative Promoter Motif Analyses Reinforce the Evolutionary Relationships Among Faustoviruses, Kaumoebavirus, and Asfarvirus

**DOI:** 10.3389/fmicb.2018.01041

**Published:** 2018-05-23

**Authors:** Graziele P. Oliveira, Isabella L. M. de Aquino, Ana P. M. F. Luiz, Jônatas S. Abrahão

**Affiliations:** Laboratório de Vírus, Departamento de Microbiologia, Instituto de Ciências Biológicas, Universidade Federal de Minas Gerais, Belo Horizonte, Brazil

**Keywords:** faustovirus, kaumoebavirus, asfarvirus, promoter, core-genes

## Abstract

Putative promoter motifs have been described in viruses belonging to the nucleocytoplasmic large DNA viruses (NCLDVs) group; however, few studies have been conducted to search for promoter sequences in newly discovered amoebal giant viruses. Faustovirus and kaumoebavirus are two *Asfarviridae*-related giant viruses belonging to the NCLDVs group. The phylogenetic relationships among these viruses led us to investigate if the promoter regions previously identified in the asfarvirus genome could be shared by its amoebal virus relatives. Previous studies demonstrated the role of A/T-rich motifs as promoters of asfarvirus. In this study, we reinforce the importance of A/T rich motifs in asfarvirus and show that the TATTT and TATATA motifs are also shared in abundance by faustovirus and kaumoebavirus. Here, we demonstrate that TATTT and TATATA are mostly present in faustovirus and kaumoebavirus genomic intergenic regions (IRs) and that they are widely distributed at 0 to -100 bp upstream to the start codons. We observed that putative promoter motifs are present as one to dozens of repetitions in IRs of faustovirus, kaumoebavirus, and asfarvirus, which is similar to that described previously for marseilleviruses. Furthermore, the motifs were found in most of the upstream regions of the core genes of faustovirus, kaumoebavirus, and asfarvirus, which suggests that the motifs could already be present in the ancestor of these viruses before the irradiation of this group. Our work provides an in-depth analysis of the putative promoter motifs present in asfarvirus, kaumoebavirus, and faustovirus, which reinforces the relationship among these viruses.

## Introduction

The nucleocytoplasmic large DNA viruses (NCLDVs) group has been expanding in number and diversity since the discovery of the *Acanthamoeba polyphaga mimivirus* (APMV) in 2003 (La Scola et al., 2003; Boyer et al., 2009; Pagnier et al., 2013; Philippe et al., 2013; Legendre et al., 2014, 2015). Faustovirus and kaumoebavirus are two Asfarvirus-related giant viruses that belong to the NCLDV group (Reteno et al., 2015; Bajrai et al., 2016). Faustoviruses strains were first isolated in France and Senegal on *Vermamoeba vermiformis* (Reteno et al., 2015). In addition, the detection of a faustovirus-like virus has already been described in hematophagous arthropods and in their animal hosts and in human samples (Temmam et al., 2015). Faustovirus has a double-stranded DNA with a circular shape genome (except for the Faustovirus Liban strain for which a linear genome was suggested) with approximately 466 kbp encoding 451 predicted proteins. These viruses form 200 nm particles (icosahedral symmetry) with a unique structure and two protein shells (Reteno et al., 2015; Benamar et al., 2016; Klose et al., 2016; Louazani et al., 2017). Its unique architecture combined with a large number of introns and exons found in gene coding the major capsid protein were associated with the virus’s ability to adapt to new environments or hosts (Klose et al., 2016). About two-thirds of the faustovirus genes are ORFans (ORFs with no detectable homolog). Furthermore, paralogous genes represent 19% of the faustovirus gene complement (Boyer et al., 2010; Rinke et al., 2013; Reteno et al., 2015). Similar to marseilleviruses and other giant viruses that infect amoebas, the faustoviruses exhibit a high level of genomic mosaicism, which were identified as proteins hits with other giant viruses, bacteria, eukaryotes, archaea and phages, and that best matches with proteins identified from African swine fever virus (ASFV) (Boyer et al., 2009; Reteno et al., 2015). A phylogenetic analysis revealed a relationship between faustoviruses and asfarvirus, which suggests a shared origin (Reteno et al., 2015; Benamar et al., 2016).

Currently, ASFV is a single member of the *Asfarviridae* family and *Asfivirus* genus. ASFV is a large (∼200 nm), icosahedral, and enveloped virus that infects members of the Suidae family (Tulman et al., 2009). Its genome is composed of a linear dsDNA molecule of approximately 170 kbp that encodes approximately 150 ORFs (Yáñez et al., 1995). ASFV encodes its own RNA pol and ASFV genes are transcribed by its enzyme (Kuznar et al., 1980; Salas et al., 1988). The asfarvirus intergenic genomic regions are rich in A/T sequences and the characterization of the promoter motifs for the late asfarvirus gene B646L coding the major capsid protein showed the importance in gene expression of A/T rich regions containing TATTT and TATATA motifs, wherein the sequence located at -2 to +2 appears to be a more critical region for B646L promoter activity (Yáñez et al., 1995; García-Escudero and Viñuela, 2000; Rodríguez and Salas, 2013). Furthermore, biological experiments involving genetic deletions, linker scan substitutions and point mutations in these genomic regions revealed that the replacement of the A/T-rich region by G/C residues strongly reduced the transcription rate and demonstrated the importance of this sequence for viral transcription (Rodríguez et al., 1996; García-Escudero and Viñuela, 2000; Rodríguez and Salas, 2013).

Contributing to the expansion of the NCLDV group, a new giant virus was isolated in sewage water from Saudi Arabia and named Kaumoebavirus (Bajrai et al., 2016). The kaumoebavirus have a morphology (∼250 nm icosahedral capsids) and genome (350,731 bp double-stranded DNA genome coding 465 genes) similar to faustoviruses. Furthermore, this giant virus was isolated on the same amoeba as faustovirus (*Vermamoeba vermiformis*) and the best matches to its proteins are to faustoviruses and asfarviruses. Accordingly, phylogenetic analysis showed that kaumoebavirus is a distant relative of faustoviruses and asfarviruses (Bajrai et al., 2016).

Promoter motifs have been described in viruses that belong to the NCLDV group such as in poxviruses, iridoviruses, phycodnaviruses, ascovirus, and asfarvirus (García-Escudero and Viñuela, 2000; Suhre et al., 2005; Nalçacioǧlu et al., 2007; Fitzgerald et al., 2008; Salem et al., 2008; Legendre et al., 2010; Yang et al., 2011; Oliveira et al., 2017a). However, little is known about the promoter sequences in giant viruses since the putative promoter motif has been described only for the mimivirus and marseillevirus families (Suhre et al., 2005; Oliveira et al., 2017b). Here, we reinforce the importance of A/T rich motifs (TATTT and TATATA) in asfarvirus and for the first time identified those motifs as supposed sequences that can function as putative promoters in faustovirus and kaumoebavirus. Furthermore, in conjunction with core gene analyses, we suggest that TATTT and TATATA motifs could be present in the ancestor of faustovirus, kaumoebavirus, and asfarvirus.

## Materials and Methods

### Motif Analyses

The genomic sequences of faustoviruses, kaumoebavirus, and asfarvirus were analyzed: seven faustovirus strains (faustovirus strain E12-the prototype member, faustovirus D5a, faustovirus D5b, faustovirus D6, faustovirus E23, faustovirus E24, and faustovirus ST1); kaumoebavirus (kaumoebavirus isolate Sc) and asfarvirus (ASFV strain BA71V). The genome sequences used here are available in GenBank under accession numbers KJ614390.1; KU702950.1; KU702949.1; KU702951.1; KU702952.1; KU702948.1; LT839607.1; NC_034249.1; and NC_001659.2. The intergenic regions (IRs) of these viruses were obtained using Artemis software (Rutherford et al., 2000). A total of 489 genomic IR were obtained from the faustovirus E12 genome (274 in the positive strand and 215 in the negative strand), 487 from the faustovirus D5a genome (272 in the positive strand and 215 in the negative strand), 484 from the faustovirus D5b genome (208 in the positive strand and 276 in the negative strand), 486 from the faustovirus D6 genome (271 in the positive strand and 215 in the negative strand), 492 from the faustovirus E23 genome (277 in the positive strand and 215 in the negative strand), 492 from the faustovirus E24 genome (275 in the positive strand and 217 in the negative strand), 470 from the faustovirus ST1 genome (272 in the positive strand and 198 in the negative strand), 423 from the kaumoebavirus genome (260 in the positive strand and 163 in the negative strand), and 141 from the asfarvirus genome (70 in the positive strand and 71 in the negative strand). The accession numbers and the IR data are shown in **Table 1**. The total number of IR that we considered in the analysis were 485 for faustovirus E12, 484 for faustovirus D5a, 478 for faustovirus D5b, 480 for faustovirus D6, 487 for faustovirus E23, 488 for faustovirus E24, 467 for faustovirus ST1, 417 for kaumoebavirus, and 135 for asfarvirus (the IR that contained less than 8 bp were not considered). The search for motifs was performed in IR and in coding sequences (CSs) by manual analysis for all of the viral species mentioned above. The distance between the motifs and the start codon was calculated for faustovirus E12, kaumoebavirus, and asfarvirus. The graphs were generated using GraphPad Prism version 7.00 (GraphPad Software).

**Table 1:** Accession numbers and intergenic regions data of the faustoviruses, kaumoebavirus, and asfarvirus strains.

	Strains	Accession number	Number of intergenic regions	
			Positive strand	Negative strand
Faustoviruses	E12	KJ614390.1	274	215
	D5a	KU702950.1	272	215
	D5b	KU702949.1	208	276
	D6	KU702951.1	271	215
	E23	KU702952.1	277	215
	E24	KU702948.1	275	217
	ST1	LT839607.1	272	198
Kaumoebavirus	Sc	NC_034249.1	260	163
Asfarvirus	BA71V	NC_001659.2	70	71

### Phylogenetic Analyses

Complete DNA polymerase B protein sequences were aligned using the MUSCLE program (Edgar, 2004). Evolutionary analyses were conducted in MEGA7 (Kumar et al., 2016). The phylogenetic analyses were inferred by using the maximum likelihood method based on the JTT matrix-based model (Jones et al., 1992). The percentage of trees in which the associated taxa clustered together is shown next to the branches. The bootstrap values above 800 are shown. The analysis involved 41 DNA polymerase B amino acid sequences. The accession numbers are shown in the phylogenetic tree.

### Core-Gene Analysis

The Proteinortho tool (Lechner et al., 2011) was used to define the strict core of orthologs shared among the Faustovirus E12 strain, the Kaumoebavirus isolate Sc and ASFV strain BA71V. The core of orthologs shared among the Faustovirus E12 strain and the Kaumoebavirus isolate Sc and among were defined, among the Kaumoebavirus isolate Sc and the ASFV strain BA71V and among the Faustovirus E12 strain and the ASFV strain BA71V. The thresholds for the *e*-value, identity and coverage of amino acid sequences were 10^-5^, 25 and 50%, respectively. The distribution of the motif sequences was evaluated in upstream regions of the core genes by manual analyses.

## Results

### A/T Rich Promoters Motifs of the Asfarvirus in Genomic Intergenic Regions of the Faustovirus and Kaumoebavirus

**FIGURE 1:**
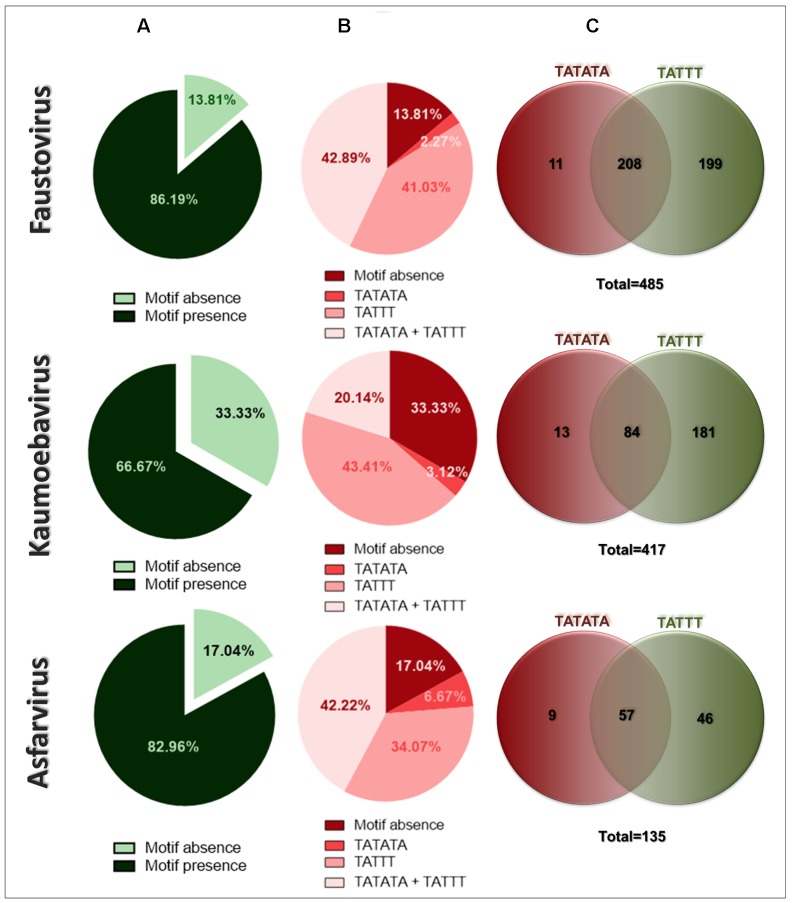
Percentage of intergenic regions (IRs) containing the TATTT and TATATA motifs: faustovirus, kaumoebavirus, and asfarvirus. **(A)** The total distribution of TATTT and TATATA motifs in the IRs. **(B)** The differential distribution of only the TATTT or TATATA motifs of both motifs in the same IR. **(C)** A Venn diagram representing the absolute number of IRs containing only TATTT or TATATA motifs and of those with both motifs.

The search for repeated motifs in the IR of faustovirus and kaumoebavirus revealed the presence of regions containing A/T rich motifs, which play a promoter role in asfarvirus. To evaluate the distribution of motifs in the IR of faustovirus, kaumoebavirus, and asfarvirus, we searched and counted the number of IR that present any of the motifs. We observed that the motifs occurred in 418 of the 485 (86.19%) genomic IR of the faustovirus. In kaumoebavirus and asfarvirus, the motifs were found in 278 of 417 (66.67%) genomic IR and 112 of 135 (82.96%) genomic IR, respectively (**Figure 1A**). Moreover, the motifs were found alone (TATTT or TATATA) or in pairs (TATTT/TATATA) in the same IR of faustovirus, kaumoebavirus, and asfarvirus. A total of 41.03% (199/485) of the IR of the faustovirus presented only the TATTT motif, 2.27% (11/485) of the faustovirus IR presented only the TATATA and 42.89% (208/485) of the faustovirus IR presented both motifs (**Figures 1B,C**). In kaumoebavirus, 43.41% (181/417) of the IR presented only TATTT, 3.12% (13/417) of the IR only presented TATATA and 20.14% (84/417) presented both motifs (**Figures 1B,C**). In asfarvirus, 34.07% (46/135) of the IR presented only TATTT, 6.67% (9/135) of the IR only presented TATATA and 42.22% (57/135) presented both motifs (**Figures 1B,C**).

### Distribution of the TATTT and TATATA Motifs and Its Localization Upstream of the ATG

**FIGURE 2:**
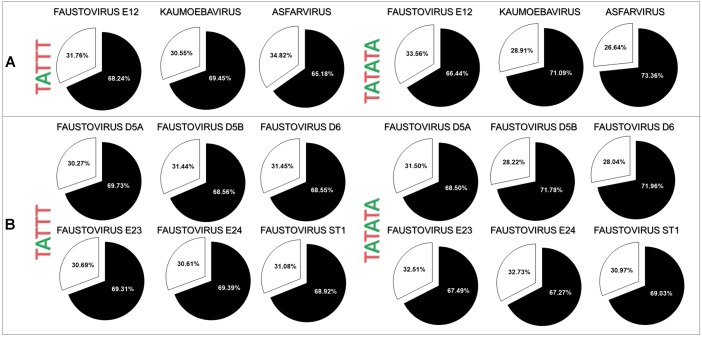
Occurrence of the TATTT/TATATA motifs in genomic IRs or coding sequences (CSs) of faustovirus, kaumoebavirus, and asfarvirus genomes. **(A)** The prevalence of the TATTT/TATATA motifs in faustovirus E12, kaumoebavirus, and asfarvirus genomes and in other faustovirus strains **(B)**. The distribution of the TATTT/TATATA motifs in genomic IRs and in CSs is represented by a circle with black and white colors, respectively.

**FIGURE 3:**
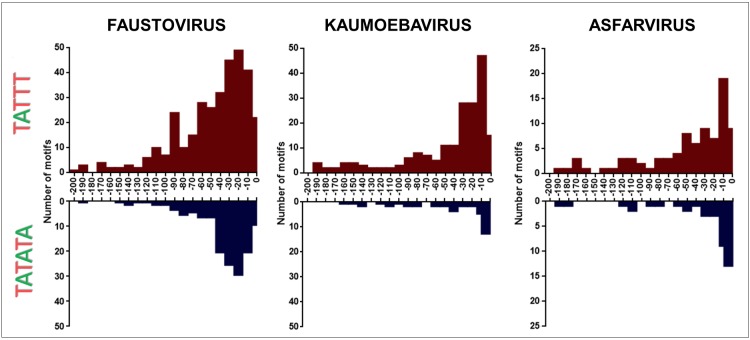
Motif distance at –200 bp upstream of the ATG start codon. The bar graphs represent the distribution –200 bp upstream of the ATG start codon to the TATTT motif and to the TATATA motif in faustovirus, kaumoebavirus, and asfarvirus.

A search and quantification of the motifs TATTT and TATATA was conducted in the genomic IR and in CSs of the strains of faustoviruses, kaumoebavirus, and asfarvirus. A total of 2608 (68.24% of the total motifs) TATTT motif copies were found in the IR of the faustovirus E12 strain, 1132 (69.45%) TATTT motif copies were found in the IR of the kaumoebavirus and 865 (65.18%) TATTT motif copies were found in the IR of the asfarvirus. Significantly less copies were found of this motif in the CS: 1214 (31.76%) TATTT motif copies were found in the CS of the faustovirus, 498 (30.55%) TATTT motif copies were found in the CS of the Kaumoebavirus and 462 (34.82%) TATTT motif copies were found in the CS of the asfarvirus (**Figure 2A**). Although it is present in a smaller quantity if compared to the TATTT motif, a higher prevalence in the IR was also demonstrated for the TATATA motif, which was found 590 (66.44%) TATATA motif copies in the IR of the faustovirus E12 strain and only 298 (33.56%) in the CS, 150 (71.09%) TATATA motif copies in the IR of the kaumoebavirus and only 61 (28.91%) in the CS. For asfarvirus were found 157 (73.36%) TATATA motif copies in the IR and only 57 (26.64%) times in the CS (**Figure 2A**). We expanded this analysis to other faustovirus isolates, and we observed similar results (**Figure 2B**). In the faustovirus D5a strain, 69.73% of the total TATTT motifs were found in their IR; in the faustovirus D5b, D6, E23, E24 and ST1 strains, this motif was found in 68.56, 68.55, 69.31, 69.39, and 68.92% of the IR, respectively. TATATA was found in 68.50, 71.78, 71.96, 67.49, 67.27, and 69.03% of the TATATA motifs in the IR of the faustovirus strains D5a, D5b, D6, E23, E24, and ST1, respectively. The localization relative to the start codon was identified for both motifs in faustovirus, kaumoebavirus, and asfarvirus. The TATTT and TATATA motifs are located mainly up to -100 base pairs (bp) upstream of ATG (**Figure 3**). More specifically, the TATTT motifs are located mainly at 0 to -100 bp upstream from the ATG and the TATATA motifs are located mainly at 0 to -50 bp of faustovirus genes, while in kaumoebavirus and asfarvirus the TATTT and TATATA motifs are located mainly at 0 to -50 bp and 0 to -20 bp from the start codon, respectively (**Figure 3**).

### Repetitions of the TATTT and TATATA Motif in Genomic Intergenic Regions

**FIGURE 4:**
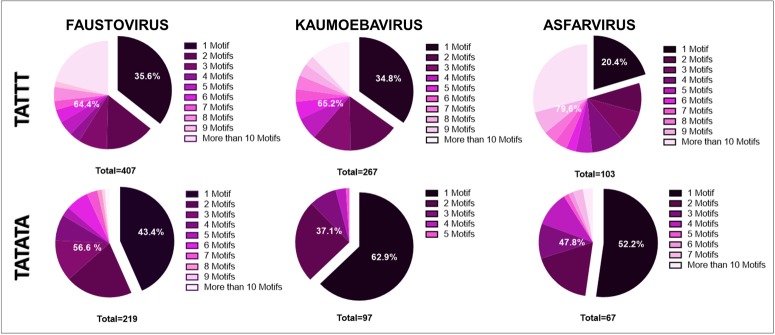
Number of repetitions of the motifs in each IR. The number of repetitions of the TATTT and TATATA motif in faustovirus, kaumoebavirus, and asfarvirus.

The quantification of the motifs present in each genomic IR showed that 35.6% (145/407) of the faustovirus IR have only one copy of the TATTT motif and 64.4% (262/407) present more than one copy of the same motif (**Figure 4**). In kaumoebavirus, only one copy of the TATTT motif was found in 34.8% (93/267) of the IR and more than one TATTT motif copy was observed in 65.2% (174/267) of the IR (**Figure 4**). A similar profile was observed in asfarvirus since 20.4% (21/103) of its IR presents only one copy of the TATTT motif and 79.6% (82/103) show more than one copy of the motif (**Figure 4**). Regarding the TATATA motif, the occurrence of one motif copy in 43.4% (95/219), 62.9% (61/97), and 52.2% (35/67) of the genomic IR of the faustovirus, kaumoebavirus, and asfarvirus was demonstrated, respectively (**Figure 4**). More than one copy of the TATATA motif was observed in 56.6% (124/219) of the faustovirus genomic IR, in 37.1% (36/97) of the kaumoebavirus genomic IR and in 47.8% (32/67) of the asfarvirus genomic IR (**Figure 4**).

### Distribution of the TATTT and TATATA Motifs at Core Genes’ Upstream Regions of Faustoviruses, Kaumoebavirus, and Asfarvirus

**FIGURE 5:**
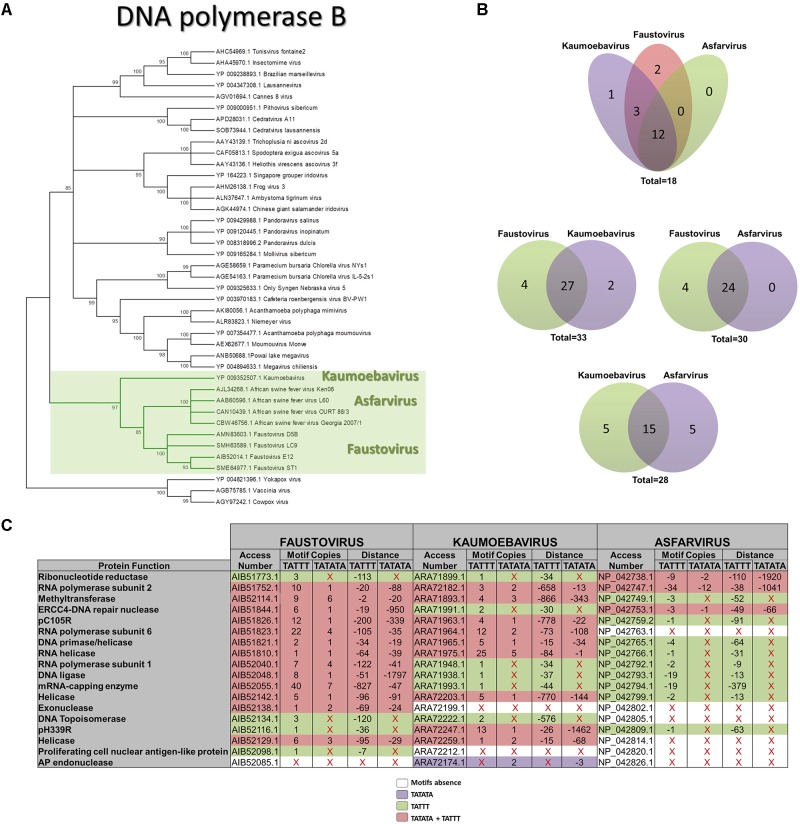
Phylogenetic tree of the B DNA polymerase and core-gene analyses. **(A)** The phylogenetic analyses using the maximum likelihood method and based on the family B DNA polymerases protein sequences from faustoviruses, kaumoebavirus, and asfarvirus strains and other representative members of the NCLDV. The accession numbers are shown in the tree. **(B)** Venn diagrams representing the presence of the motifs in the upstream regions of the core-genes. **(C)** A detailed representation of the core-genes and their functions and the presence of the motifs upstream of the core genes of faustovirus, kaumoebavirus, and asfarvirus. The number and localization of the motif relative to the start codon are demonstrated.

The phylogenetic relationship among fautovirus, kaumoebavirus, and asfarvirus was reinforced in this study by the phylogenetic reconstruction based on family B DNA polymerase (**Figure 5A**). We performed a comparative analysis of the faustovirus, kaumoebavirus, and asfarvirus complete gene set with the aim of determining the core genes among this group of viruses. Our data reveals 18 genes that are shared by these three viruses, 28 core-genes that are shared between kaumoebavirus and asfarvirus, 30 core-genes that are shared between faustovirus and asfarvirus and 33 core-genes that are shared between faustovirus and kaumoebavirus. The search by the TATTT and TATATA motif sequences in the upstream regions of these genes revealed that most of the core genes (12/18) of the faustovirus, kaumoebavirus, and asfarvirus had at least one of the motifs in their upstream region (**Figures 5B,C**). We also observed that among the 33 core-genes shared by faustovirus and kaumoebavirus, 27 present the putative promoter motifs. Considering faustovirus and asfarvirus, we observed that among their 30 core-genes, 24 shared the motifs, and among the 28 core-genes shared by kaumoebavirus and asfarvirus, 15 present the putative promoter motifs (**Figure 5B**). This represents the presence of at least one of the motifs in 94.4, 88.9, and 66.7% at the IR upstream to the core genes of faustoviruses, kaumoebaviruses and asfarviruses, respectively. Furthermore, it represents a higher perception of motif presence in core genes than that observed for the rest of the genome of faustovirus (86.2%) and kaumoebavirus (66.7%); however, for asfarviruses, the perception of core genes presenting at least one of the promoter motifs is smaller than the rest of the genome (82.9%).

## Discussion

Genomic studies have revealed that faustovirus, kaumoebavirus, and asfarvirus are phylogenetically related (Reteno et al., 2015; Bajrai et al., 2016). In this study, the search for repeated motifs in the IR of faustovirus and kaumoebavirus revealed the wide distribution of the TATTT and TATATA motifs that play a promoter role in asfarvirus (Rodríguez et al., 1996; García-Escudero and Viñuela, 2000; Rodríguez and Salas, 2013). Given the phylogenetic proximity demonstrated between faustovirus, kaumoebavirus, and asfarvirus and associated with the wide distribution of the same motifs in the IR of these viruses, we suggest that the regions containing the motifs TATTT and TATATA can also play an important role in the gene expression of faustovirus and kaumoebavirus. It has already been demonstrated that ASFV encodes its own RNA pol and ASFV genes are transcribed by its enzyme (Kuznar et al., 1980; Salas et al., 1988). The RNA pol sequences were also predicted in the genome of faustovirus and kaumoebavirus. Although studies have not been conducted to demonstrate the actual activity of RNA polymerase in the expression of their genes, we believe that this is the case, which reinforces the presence of a similar promoter sequence in these viruses since their RNA polymerase subunits constitute core genes in these viruses and reflect a common origin. Previous studies showed a wide distribution of putative promoter motif in the IR of mimivirus, and half of the genes present the AAAATTGA in their upstream region. Similar results were described for marseilleviruses, and more than half of the genes demonstrated the presence of the motif promoter in its upstream regions (Suhre et al., 2005; Oliveira et al., 2017b). The analyses showed that the putative promoter motifs were found alone (TATTT or TATATA) or in pairs (TATTT/TATATA) in the same IR of faustovirus, kaumoebavirus, and asfarvirus. As previously demonstrated for asfarvirus, these data may suggest that the motifs TATTT and TATATA can act simultaneously in a given IR of faustovirus and kaumoebavirus (Rodríguez and Salas, 2013). The distribution analyses of the motifs TATTT and TATATA demonstrated a significantly higher occurrence of the TATTT and TATATA motifs in the genomics IR compared to CS for all of the analyzed viruses. Furthermore, the TATTT and TATATA motifs are located mainly at 0 to -100 base pairs upstream of ATG follow a similar promoter localization profile demonstrated for other organisms. The TATA Box is a classical promoter element of eukaryotes that are located in IRs approximately at -25 to -30 bp upstream of the transcription start site (Haberle and Lenhard, 2016). Bacterial genomes present short conserved motifs that are also rich in A/T, which are located approximately -10 and -35 bp upstream of the transcription start site (Browning and Busby, 2004). Regions rich in A/T at the 150 bp position upstream of ATG are predicted promoter sequences for other NCLDV members, such as poxviruses, asfarviruses, phycodnaviruses, iridoviruses, and for the mimiviruses, which are the first discovered amoebal giant viruses (García-Escudero and Viñuela, 2000; Suhre et al., 2005; Nalçacioǧlu et al., 2007; Fitzgerald et al., 2008; Salem et al., 2008; Legendre et al., 2010; Yang et al., 2011). Furthermore, the same localization profile was also observed for marseillevirus, which is a giant virus that belongs to the NCLDV group (Oliveira et al., 2017b). Despite the fact that the importance of the specific positions of the motifs have already been demonstrated for asfarvirus gene expression, the role of the motifs in different IR positions for faustovirus and kaumoebavirus should not be discarded. For this reason, we considered more localization possibilities in our analysis. In this study was showed that the IRs of the faustovirus, kaumoebavirus, and asfarvirus display multiple repetitions of the TATTT and TATATA motifs, but it is not the first time that multiple copies of putative promoter motifs’ have been described. A recent study suggested that the promoter sequence repetition in the marseillevirus genome can be associated with lateral-gene-transfer (LGT) events (Oliveira et al., 2017b). It has been suggested that viruses with large dsDNA genomes can evolve by gene acquisition from the genomes of cellular organisms and their hosts. Indeed, many genes of these viruses show high levels of sequence similarity to their cellular homologs, which is apparently indicative of relatively recent acquisition by the viral genomes (Bugert and Darai, 2000). Previous works suggest that LGT events are common among NCLDV (Boyer et al., 2009). In this way, it is noteworthy that both faustovirus and kaumoebavirus exhibit a high level of genomic mosaicism that can be associated with LGT events (Reteno et al., 2015; Bajrai et al., 2016). Furthermore, paralogous genes represent 19% of the faustovirus gene complement and motif repetitions may be related to genetic duplications in the genome of these viruses.

The core genes’ analysis revealed that most of the orthologous genes shared among faustovirus, kaumoebavirus, and asfarvirus show the same predicted motif sequence. These data suggest that the motif here described sequences could be present in the ancestor of faustovirus, kaumoebavirus, and asfarvirus. There is still much to learn about the structure of the promoters and the identity of transcription factors, which regulate the gene expression in giant viruses; however, with each new analysis, we can add a brick to this construction of knowledge.

## Author Contributions

GO, IdA, and AL performed the analyses. JA designed the study. GO, IdA, and JA wrote the manuscript. All authors approved the manuscript.

## Conflict of Interest Statement

The authors declare that the research was conducted in the absence of any commercial or financial relationships that could be construed as a potential conflict of interest.
